# Structural information in therapeutic peptides: Emerging applications in biomedicine

**DOI:** 10.1002/2211-5463.13847

**Published:** 2024-06-14

**Authors:** Valentín Iglesias, Oriol Bárcenas, Carlos Pintado‐Grima, Michał Burdukiewicz, Salvador Ventura

**Affiliations:** ^1^ Institut de Biotecnologia i de Biomedicina and Departament de Bioquímica i Biologia Molecular Universitat Autònoma de Barcelona Barcelona Spain; ^2^ Clinical Research Centre Medical University of Białystok Białystok Poland; ^3^ Institute of Advanced Chemistry of Catalonia (IQAC), CSIC Barcelona Spain

**Keywords:** antimicrobial peptides, peptide drug development, peptide structure, peptides, therapeutic peptides, translational medicine

## Abstract

Peptides are attracting a growing interest as therapeutic agents. This trend stems from their cost‐effectiveness and reduced immunogenicity, compared to antibodies or recombinant proteins, but also from their ability to dock and interfere with large protein–protein interaction surfaces, and their higher specificity and better biocompatibility relative to organic molecules. Many tools have been developed to understand, predict, and engineer peptide function. However, most state‐of‐the‐art approaches treat peptides only as linear entities and disregard their structural arrangement. Yet, structural details are critical for peptide properties such as solubility, stability, or binding affinities. Recent advances in peptide structure prediction have successfully addressed the scarcity of confidently determined peptide structures. This review will explore different therapeutic and biotechnological applications of peptides and their assemblies, emphasizing the importance of integrating structural information to advance these endeavors effectively.

## Graphical abstract

Peptides are increasingly popular as therapeutic agents due to their cost‐effectiveness, low immune response, and high specificity. Many tools study peptides as linear chains, ignoring crucial structural details that affect their properties. Recent advances in peptide structure prediction have improved our understanding. This review highlights the importance of integrating structural information to advance the therapeutic and biotechnological use of peptides.

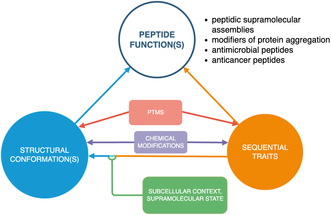

**Abbreviations**
ACP: Anticancer peptide
AMP: Antimicrobial peptide
EMA: European Medicines Agency
FDA: U.S. Food and Drug Administration
IR: Insulin receptor
MD: Molecular Dynamics simulations
ML: Machine learning
NMR: nuclear magnetic resonance
PEG: polyethylene glycol
PPI: Protein–protein interaction
SPPS: Solid‐phase peptide synthesis

##  

Peptides are short biopolymers of proteinaceous nature [1, 2] which serve multifaceted and crucial roles in living organisms, acting as messengers in regulation, neurotransmission, cell signaling, and structural or immune‐response elements, as well as acting as toxins and venoms [3, 4, 5, 6, 7]. This functional diversity arises from the broad conformational landscape these peptides can populate, which ranges from fully unstructured to fully structured ensembles. Some peptides are heavily influenced by their environment, undergoing conformational transitions upon binding to protein or lipid partners [8] or in response to environmental conditions [9]. To study the conformational properties of peptides, nuclear magnetic resonance (NMR) spectroscopy is often used, which captures an ensemble of conformations and reflects the molecular flexibility and thermal motion of the molecule. Alternative methods, like X‐ray crystallography or cryogenic electron microscopy, often require peptides to be bound to larger protein structures, constraining the accessible conformational space, potentially skewing the peptide structure from this in solution.

The conformation of many peptides is inherently dynamic, a consequence of their low secondary and tertiary structure content. Despite the efforts of obtaining structures that faithfully recapitulate the conformation of peptides in solution, the experimental conditions used to solubilize or perform structural determinations often impact the resulting architecture. This might artificially favor local interactions over those with water and, as a consequence, exaggerates the detected secondary structure [10, 11]. As a consequence, unstructured peptides in aqueous environments, existing as random coils or extended conformations [12], are often overlooked and underrepresented in the dataset of available structures.

This inherent instability makes native peptides proteolytically sensitive, accelerating *in vivo* clearance. Natural biopeptides, however, use various strategies to avoid rapid turnover, exploiting post‐translational modifications such as glycosylation, amidation, halogenation, phosphorylation, incorporation of unconventional D‐amino acids, or cyclization [13]. These protective modifications can heavily influence peptide's structure and are often incorporated in artificial peptides.

Peptidic chains can be arranged in cyclic ring structures by linking the N‐ and C‐terminal ends by an amide bond (also known as “head‐to‐tail”), through covalent bonding with an amino acid side chain (“backbone‐to‐side‐chain”) or by side‐chain‐to‐side‐chain bonds (such as disulfide, thioether, ether or lactones) [14]. Their circular disposition offers several advantages compared to their linear counterparts, including higher membrane permeability, increased thermodynamic stability, and protection against exopeptidases [15]. For instance, cyclic peptides were found to selectively inhibit anti‐apoptotic (pro‐survival) BCL‐2 and BCL‐XL proteins, highly overexpressed in leukemia. The spatial disposition of the peptides' residues allowed them to bind with nanomolar affinity, comparable to approved pharmaceuticals like Venetoclax [16].

Beyond their inherent structural diversity, another remarkable feature of many peptides is their ability to undergo conditional folding upon interaction with target ligands or when encountering specific solvent conditions. For example, Histatin‐5, an initially disordered antimicrobial peptide found in saliva, gains compactness when coordinating Zn^2+^ and adopts an alpha‐helical structure in lipid vesicles, each transition impacting Histatin‐5's antimicrobial potency [17, 18].

## Bioinformatic tools assist peptide structure and function prediction

### Peptide function prediction

Peptides' have a large potential in pharmaceutical drug development and other industrial applications; accordingly, multiple bioinformatic tools have been developed to predict peptide function from sequential information. *In silico* approaches appear as cost and time‐efficient ways for initial screening and preselection of candidates from large established peptide libraries or from high‐throughput sequencing endeavors for subsequent experimental validation of peptides.

We previously surveyed >140 tools for the prediction of peptide function, with most tools focusing on predicting a single bioactive function [19]. Most of these algorithms were devoted to identifying anticancer (ACP) and antimicrobial peptides (AMP). AMPScanner V2 was the first tool to apply a deep learning model [20]. AmpGram [21] and ampir [22] achieve high performance using machine learning approaches and are devised for proteome‐wide analyses. Macrel follows this path as it is optimized for genome and metagenome screenings, allowing the input of DNA sequences [23]. Moreover, Macrel can predict AMPs hemolytic potential. Ensemble‐AMPPred concatenates various machine learning prediction models to identify AMPs [24]. Finally, DBAASP combines an antimicrobial peptide database with prediction tools for the antimicrobial activity of peptides, along with modeling their cytotoxicity against mammalian cells [25, 26]. Notably, this prediction model can not only differentiate between antibacterial, antifungal, and antiviral activities, but has been trained to consider the biocide potential for specific microbial species and strains.

**Fig. 1:**
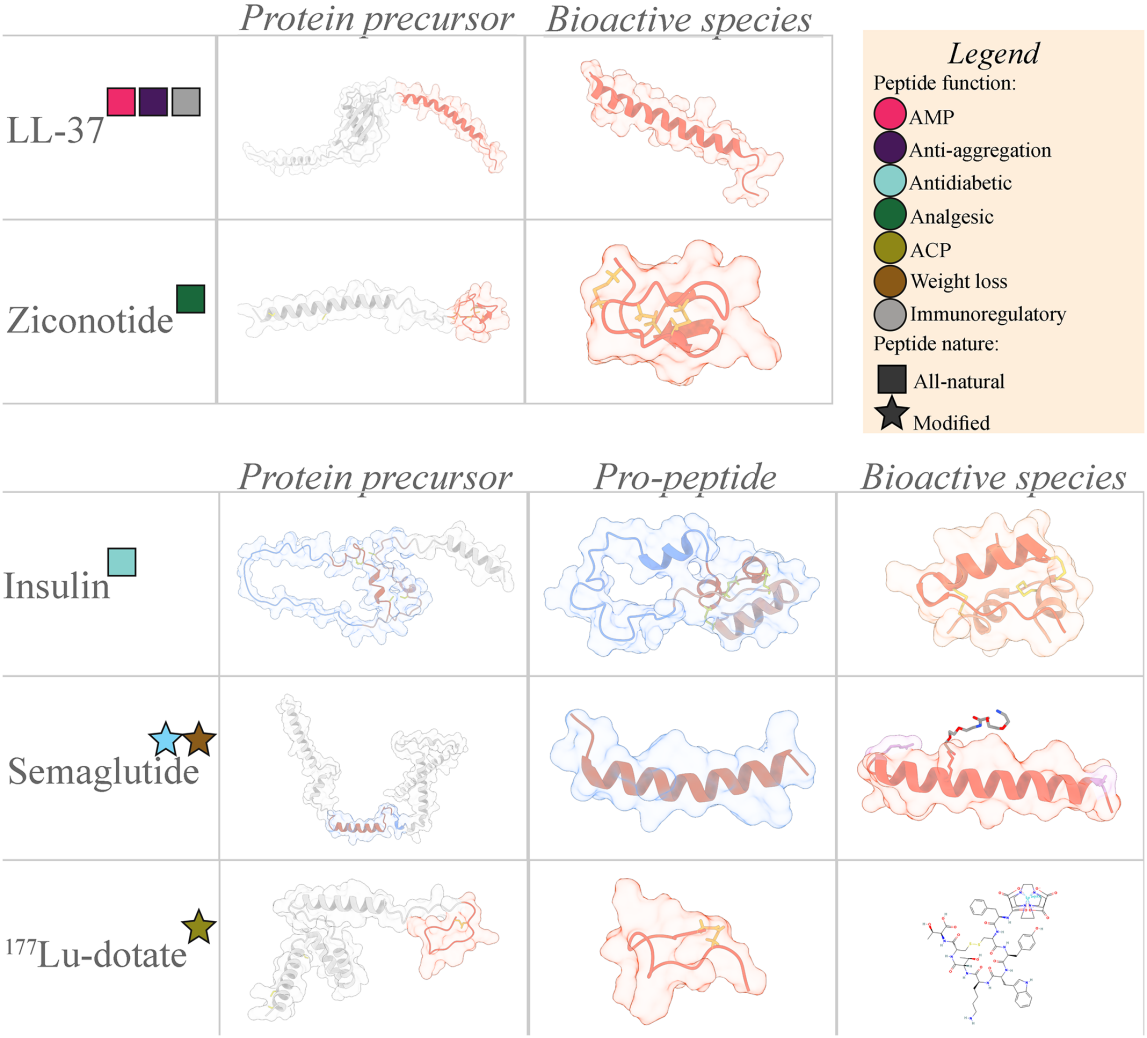
Representation of the multiple structures of five therapeutic peptides. To highlight the multiple steps peptides can undergo until reaching the bioactive species, 1–2 precursor species are presented. Residues pertaining to the protein precursor (structures obtained from the AlphaFold database [121]) are colored in gray. For those peptides with existing pro‐peptidic species, their residues have been colored in blue. The residues involved in the bioactive species are highlighted in orange color. Peptides have been subdivided between natural (squares) or modified (stars), with their specific activities presented in the figure legend. In the case of semaglutide, the stearic diacid is represented in the structure (bound to Lys26), and the modified groups (8 and 34 replaced by alpha‐aminobutyric acid and arginine) have been highlighted in violet color. With regards to ^177^Lu‐Dotatate, the smiles representation is chosen, as the magnitude of the non‐amino acidic part precluded the inclusion of a protein‐like three‐dimensional representation of the peptide. Structure sources (ordered from left to right): LL‐37: AFDB‐P49913 (AlphaFold DB), 2K6O (PDB); Ziconotide: AFDB‐P05484 (AlphaFold DB), 1MVI; Insulin: AFDB‐P01308 (AlphaFold DB), 2KQP (PDB), 6O17 (PDB); Semaglutide: AFDB‐P01275 (AlphaFold DB), 3IOL (PDB), 7KI0 (PDB); ^177^Lu‐Dotatate: AFDB‐P61278 (AlphaFold DB), 2MI1, SMILES representation.

Single‐function peptide predictors can understate peptides' potential, as multiple activities can be carried out by the same molecular entity (Fig. 1). Thus, different tools are aimed at identifying multiple peptide functionalities. CancerGram can distinguish between anticancer and antibacterial peptides [27], while PPTPP predicts antibacterial, anticancer, anti‐inflammatory, or antiviral functions relevant to peptide therapeutics [28]. MLBP predicts anticancer, antidiabetic, antihypertensive, anti‐inflammatory, and antimicrobial functions [29], and Deep2Pep discovers potential antimicrobial, antihypertensive, and antihyperglycemic bioactivities [30].

All in all, peptide activity prediction entails a complex ecosystem of tools employing different rationales behind, with a focus on assisting drug discovery. Developments in machine learning classifiers spurred the development of better‐performing models and tools [19]. We expect the recent advances seen in the structural prediction of protein structures to drive a similar improvement as ML‐predictive methods can now incorporate peptide structural attributes in their classification weights.

### Peptide structure prediction

Given the relevance of peptides' conformation in their biomedical and biotechnological applications, computational methods are a suitable complement to experimental determination for obtaining insights on peptide structure [31]. The toolbox of bioinformatics strategies to predict peptide structure encompasses *de novo* folding, homology modeling, molecular dynamics simulations (MD), and machine learning (ML) based methods.

One of the first algorithms, Geocore [32], was based on a version previously used for protein folding [33]. It exploits an energy function to narrow down the conformational space toward the most probable configurations. Similarly, PEPstr, used a generalized pattern search algorithm to define the most energy‐efficient conformations [34]. This approach was later extended by PEPstrMOD, which could predict the structure of peptides with atypical amino acids, post‐translational modifications or N‐to‐C cycles [35]. One of the newest tools that also employs sampling of the conformational space is PEP‐FOLD4 [36].

MD, usually applied to model protein trajectories, can provide unique insights into the assemblies adopted by peptides, either by themselves or when binding to their partners [37]. In this way, protein‐centric coarse‐grained simulation models such as CABS‐flex can accurately predict linear and cyclic peptide structures [38]. Another tool originally intended for protein structure prediction, MELD (Modeling Employing Limited Data), can accurately estimate relative peptide‐protein binding affinities [39].

The methods based on machine learning remain relatively underused for peptide structure prediction. However, AlphaFold2 (AF2), despite being designed to work with proteins, has shown performance on par or better than the state‐of‐the‐art peptide‐specific structure prediction methods [40]. Interestingly, AF2 can accurately predict even the structure of cyclic peptides [41]. However, its performance seems limited to well‐structured peptides with a length above 40 residues [36]. The length range between 5 and 40 residues is a specialty of the machine learning model APPTEST, which uses deep neural networks and simulated annealing to predict the structure of such short linear and cyclic peptides [42].

Overall, while peptide structure prediction has reached a level of maturity where it can accurately forecast even the most intricate configurations, it is important to choose the most appropriate tool for each application carefully.

## Relationship between peptide structure and function

### The insulin case study

**Fig. 2:**
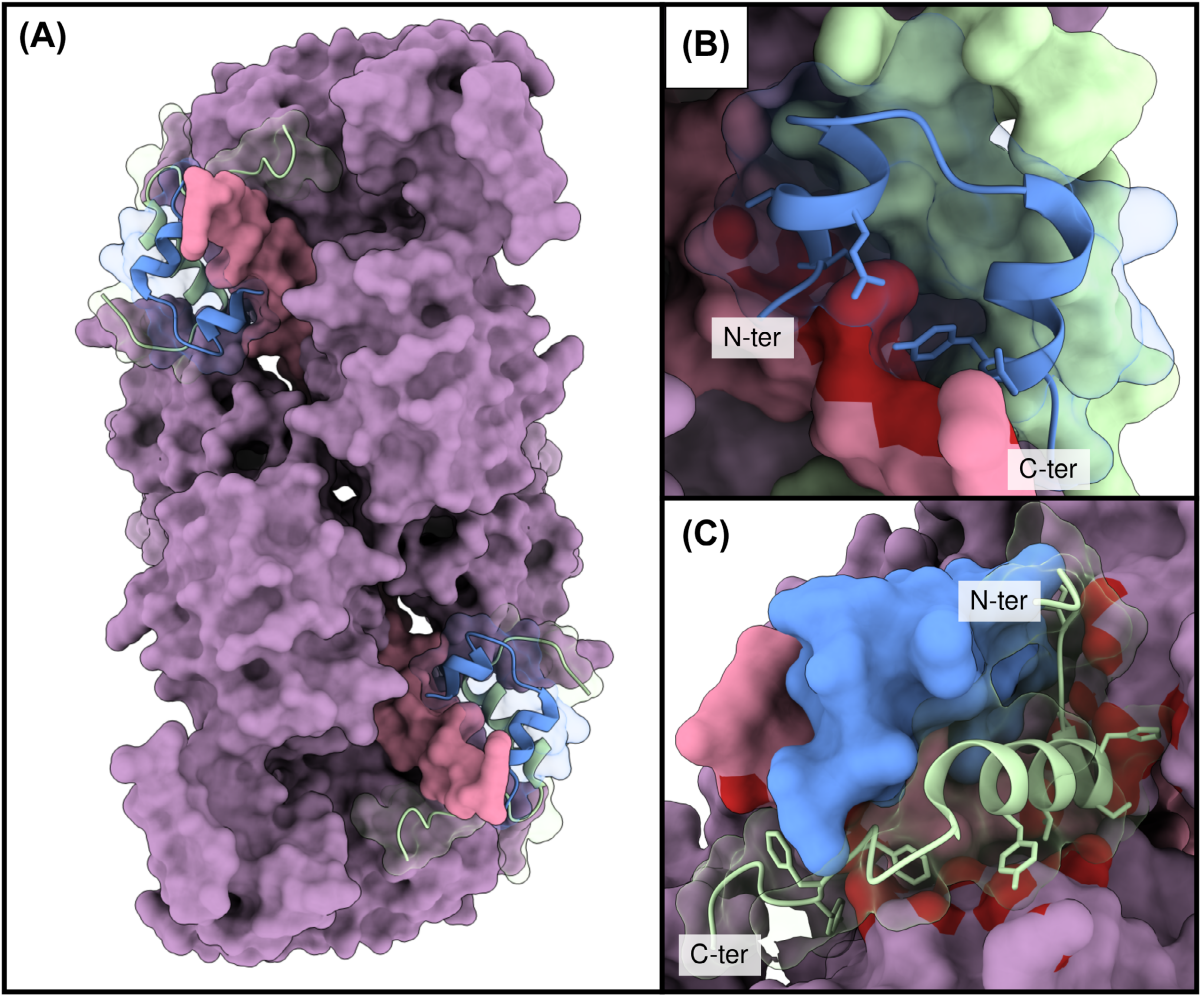
Structural analysis of insulin bound to a dimer of the α‐chain of the insulin receptor (IR). (A) Two insulin molecules are bound to the IR's α‐chain homodimer. The N terminus portion of the IR's alpha‐chain is colored violet, while the C terminus (α‐subunit C‐terminal helix, α‐CT) is colored pink. Insulin's A and B chains are colored blue and green, respectively. (B, C) Focus on the interaction surfaces. The red color represents the IR residues interacting with insulin. (B) The insulin A chain interacts most tightly with the α‐CT, especially through the peptide N‐ and C‐terminal regions. (C) The B chain establishes multiple distributed interactions, mainly with the N terminus portion of the receptor. Structure source: 6CE9 (PDB) [44].

Obtaining the native structure of insulin, the best‐known therapeutic peptide, is essential to ensure its biological activity. This peptide is synthesized as preproinsulin, which, after enzymatic removal of the signal peptide, folds and forms three disulfide bridges, being subsequently proteolyzed to yield mature insulin [43]. The active form comprises two chains, A (21 residues) and B (30 residues), which are linked by a pair of interchain disulfide bridges (A7‐B7 and A20‐B19), plus an intra‐chain disulfide bond in the A‐chain (A6‐A11) (Fig. 1). A correct peptide conformation is necessary for binding the extracellular domains of the insulin receptor (IR) (Fig. 2). This triggers autophosphorylation of the IR intracellular tyrosine kinase domains, initiating a downstream signaling cascade [44]. Administration of exogenous insulin is required to maintain glycemic control for individuals afflicted with severe insulin‐deficient and insulin‐resistant subtypes of diabetes mellitus.

The discovery of insulin and its role in the development of diabetes mellitus stands as a landmark achievement in 20th‐century medicine, awarding Frederick Banting and James Richard MacLeod the Nobel Prize in Physiology or Medicine in 1923. Just one year after its discovery, bovine and porcine insulin preparations from pancreatic extracts were already commercialized and later substituted by purified animal insulin. Immunological rejection and low activity stemming from non‐human proteins spurred the quest for human insulin. The crux of this endeavor was the generation of a correct disulfide framework, as non‐native insulin isomers could not maintain the structure required for receptor binding and activation [43]. The insulin case illustrates how structure and function are intimately connected in peptides, emphasizing that sequence information alone would have been insufficient to develop this life‐saving drug.

### Therapeutic peptides

Although historically overshadowed by compounds and larger protein‐based therapeutics such as antibodies, peptides hold vast potential as biotherapeutics owing to their nearly boundless sequential and structural diversity. When compared to organic molecules, peptide therapeutics come with certain limitations: (a) oral absorption is frequently poor (especially for linear peptides), for which the preferred administration route is usually an injection, (b) absorbed peptides can be metabolized rapidly by host proteolytic enzymes and (c) their membrane penetration capacity is generally lower (Table 1). These limitations can turn into advantages, especially in terms of toxicity, given that they are catabolized into amino acids that can be readily incorporated into cellular metabolism after acting on target molecules. Moreover, peptide biotherapeutics do not accumulate significantly in organs or tissues when compared to organic molecules [45]. Additionally, peptides' higher specificity entails lower cytotoxicity due to off‐target interactions, and their larger size (compared to non‐biological, chemical entities) allows the inhibition of larger protein–protein interaction (PPI) pockets [16].

**Table 1:** Differences in therapeutic products' characteristics by molecular entity.

Drug type	Classification	Preferred route of administration	Selectivity toward target	Membrane penetration	Clearance associated toxicity	Cost of production
Small molecule	Synthetics	Oral	Mid	High‐mid	Mid to high	Low
Peptide	Biologicals	Intravenous injection/Transnasal/Oral^a^	High	Generally mid‐low	Low	Mid
Protein – Antibody		Intravenous injection	High	Low	Low	High
Protein – General (ie. replacement enzyme)		Intravenous injection	High	Low	Low	High
DNA/RNA Aptamers		Intravenous injection	High	Low	Low	Low
Lipid/polymeric nanoparticle	Synthetics/biologicals	Intravenous injection/transdermal	High^b^	High	Low	High

^a^ Oral administration of modified peptide biotherapeutics such as Cyclosporine (Neoral®), Desmopressin (DDAVP®) or, most recently, Semaglutide (Rybelsus®) have shown the feasibility of oral routes for peptides

^b^ High when conjugated with targeting antibody, aptamer or similar.

Protein‐based drugs often display high affinity and selectivity for their targets but are challenging and expensive to obtain, as illustrated by the different flavors of therapeutic antibodies (monoclonal, humanized, single‐chain Fv (scFv), etc.). Compared to therapeutic proteins, peptides' shorter length endows them with distinct advantages, including lower production costs and easier structural and functional optimization. This is especially relevant for peptides under ~50 residues, which are straightforwardly manufactured by solid‐phase peptide synthesis (SPPS) technologies, offering higher control of the produced molecules over purified protein‐based drugs.

The administration of peptides is often easier than that of proteins and antibodies; in particular, for brain‐related diseases. The intranasal delivery of peptides provides distinctive advantages as it: (a) has direct access to the central nervous system without blood–brain barrier (BBB) blockade, (b) does not imply blood circulation preventing systemic effects, (c) reduces peptide degradation and (d) can be targeted to specific brain sites [46, 47]. Oral delivery of peptides entails numerous challenges, which include overcoming the highly denaturing conditions inside the gastrointestinal tract (low pH, enzymatic degradation) and a poor membrane permeability through the intestinal epithelium [48]. Nonetheless, oral formulations of chemically modified peptide biotherapeutics have already reached the market. Insulin, calcitonin and parathyroid hormone oral formulations are ongoing clinical trials [49].

PepTherDia [50] and THPdb [51] are manually curated repositories of approved therapeutic peptides. Besides therapeutic peptides, PepTherDia stores peptidic molecules used for diagnosis and THPdb longer proteins. Both databases include useful information to understand the pharmacological profile of the peptides, such as molecular target, route of administration, toxicity, metabolism, or disease for which they are indicated. Moreover, THPdb provides extra biological information about the peptides, including their hydrophobicity, isoelectric point or protein‐like 3D structure. Overall, more than 80 peptides have been approved as drugs by regulatory agencies and over 170 are undergoing clinical trials worldwide across diverse therapeutic areas, including urology, oncology, respiratory, pain relief, metabolic, cardiovascular, antibacterial, antiviral, and antimycotic applications, making all together the development of peptidic drugs a hot topic for biomedical research.

## Development and applications

### Uncovering natural therapeutic peptides

Increasing pharmacological efforts are dedicated to studying organisms from different environments in search of novel bioactive compounds. The strategy to garner, annotate, and characterize different organisms' peptides is called peptidomics [1]. These workflows rely on those used for high‐throughput genomics and proteomics. Despite their success [52, 53, 54], current pipelines in peptidomics predominantly treat peptides as linear entities, therefore omitting essential information about their structure and consequently skipping any structure–function relationship. Efforts should be devoted in this direction, since the expected annotation improvement is high.

One of the most promising sources of therapeutic peptides are marine organisms [55]. With their considerable evolutionary divergence from terrestrial counterparts, marine species have evolved peptides with differential composition and structural features [56]. One such example is Ziconotide (Prialt^®^) (Fig. 1) a natural peptide isolated from the venom of the cone snail *Conus magus* [57]. Ziconotide is a 25‐residue cyclic peptide containing six cysteine residues linked by three disulfide bridges. It exerts its analgesic effect by blocking N‐type voltage‐sensitive calcium channels. Its structure plays a major role in specific target recognition, binding and blocking of these ion channels [58, 59]. Despite its highly invasive (and economically expensive) intrathecal administration and severe side effects, Ziconotide has a high antinociceptive potency and an apparent lack of insensitivity when compared to morphine. These advantages drove the U.S. Food and Drug Administration (FDA) and the European Medicines Agency (EMA) approval. Expert panels have recommended it alongside morphine, as a first‐line intrathecal analgesic for patients suffering from chronic neuropathic pain [60].

### Synthetic modifications of therapeutic peptides

The chemical synthesis of peptides is usually preferred over biotechnological recombinant technology as it delivers more uniform products without traces of DNA, RNA, proteins, or other biological material. Additionally, it allows the straightforward addition of nonproteinogenic amino acids and the conjugation with biochemical or biophysical tags. Therapeutic peptides' pharmacological profiles can be tweaked by modifying the sequence‐associated chemical characteristics or the structurally encoded physical properties.

Industrially relevant peptides often undergo backbone modification to improve their proteolytic stability, including total or partial substitution of L‐amino acids by their D‐ counterparts, or β‐amino acids and residue methylation [61, 62, 63, 64]. Conversely, replacement of proteinogenic amino acids with non‐natural variants such as homoarginine, benzyloxy‐tyrosine, and β‐phenylalanine is preferably used to increase target binding or selectivity.

Additionally, the structural requirements for ligand‐binding can be enhanced by peptide cyclization or by stabilizing secondary structure elements. For instance, strategically positioning residues with high hydrogen bonding propensity in positions i, i + 4 and i + 7 to stabilize α‐helical conformations [65]. Recent protein engineering exercises introduced non‐natural amino acids capable of forming covalent bonds between those positions to promote α‐helix structures. On the other hand, adding D‐Proline to force a turn stabilizes antiparallel β‐hairpins and is a standard feature for peptides undergoing protein–protein interactions. Furthermore, covalently attaching a polyethylene glycol polymer (PEG) to Lysine or Cysteine residues has been successfully applied to delay proteolytic digestion by steric hindrance and to increase peptide solubility. Accordingly, PEGylated drugs can be found in more than 10 already available therapeutics to treat anemia, kidney disease, multiple sclerosis, hemophilia, and cancers, and various products are undergoing clinical trials [66].

In addition to providing stability and target specificity/affinity, peptide modifications offer an opportunity to expand the therapeutic repertory by combining different functions. ^177^Lu‐Dotatate (Lutathera^®^) is an example of a chemically modified therapeutic peptide (Fig. 1). It's a first‐in‐class medication composed of an octapeptide analog of somatostatin covalently bonded to a DOTA chelator containing ^177^Lutetium radionuclide [67]. The peptide recognizes somatostatin type 2 receptors, which are overexpressed on the surface of neuroendocrine tumor cells and releases the radioisotope into the tumor cells causing cellular death. Its application prolonged progression‐free survival for patients with advanced midgut neuroendocrine tumors for which both the EMA and the FDA approved it.

CyclicPepedia knowledge base compiles thousands of natural and synthetic cyclic peptides, including the dozens approved by the FDA and EMA [68]. While it is the most extensive cyclic peptide database in terms of structure and sequence, 3D information is still limited, as <5% of its entries are supported by structural experimental determination or by AlphaFold modeled structures.

### Peptidic supramolecular assemblies

Peptides are among the most common biological entities used for constructing self‐assembled macromolecular biomaterials. Their swift and economic abiotic synthesis provides higher customization options than their protein counterparts [69]. Moreover, compared to organic polymers, peptide 3D scaffolds have lower immunogenicity, lower cytotoxicity, do not require organic solvents, might display shear‐recovery capabilities and allow for recyclability of biomaterials.

Inspired by natural self‐assembly processes [70, 71], peptides capable of forming macromolecular assemblies have found applications in tissue engineering, providing scaffolds where cells can be attached to grow embedded in 3D matrices [72, 73]. The tissue microenvironment significantly impacts cellular functions and cell fate [74]. 3D extracellular matrix‐mimetics, like those formed by hydrogels, enable restoration of cell polarity, thereby affecting intracellular signaling, transcription and proliferation rates [75]. Specifically, hydrogels and amyloid architectures formed by peptides have been explored for tissue *de novo* engineering and regeneration as they provide different advantages: (a) controlled self‐assembly, (b) cell‐adhering capabilities, and (c) can be decorated for functional purposes or for tailoring the material properties [76]. For instance, nanofibrils formed by C‐terminal peptides from Aβ42 form thermoreversible, non‐toxic hydrogels and support attachment and spreading across diverse cell types [77]. Fibrillar assemblies of peptide Q11 (QQKFQFQFEQQ) decorated with short ligands (RGDS or IKVAV) promoted attachment, growth and spreading of HUVEC cells and showed a low immunogenic response when administered to mice [78]. Fibrils formed from TTR1 undecapeptide from transthyretin protein (TTR) were decorated with the linear RGD cell adhesion sequence or with a cyclic pentapeptide RGDfK [79], in both cases promoting cell adhesion and spreading. Finally, amyloids formed by peptides resulting from lysozyme hydrolysis, when deposited on polymeric films, promote increased attachment and proliferation compared to flat polymeric surfaces [80].

Beyond scaffolding cell growth, alternative uses of the controlled self‐assembly of peptides into highly ordered amyloids have been explored. In this way, amyloid fibers from Tau protein hexapeptide VQIVYK and derivatives were shown to capture CO_2_ [81], although their potential use in humans is compromised by their risk of seeding the aggregation of wild‐type Tau protein [82]. In a parallel pursuit, amyloids formed from artificial hexapeptides were shown to efficiently coordinate divalent metal cations and function as esterases and carbonic anhydrases, all while avoiding the danger of seeding or cross‐seeding the aggregation of natural proteins [83]. Even yet unexplored, these peptide self‐assembled catalytic fibers, with their biodegradable and innocuous nature, might find application in biomedicine, functioning as chelation agents for heavy metal poisoning or as nanoscopic enzymes [84, 85, 86]. Additionally, stimuli‐responsive catalytic nanomaterials have been developed recently with small peptides. By leveraging combinations of His and Tyr residues within hexa to nonapeptides, the assembly reaction can be triggered or reversed merely by adjusting the solution pH within physiological ranges [87, 88].

### Modifiers of protein aggregation

Peptides designed to impede the progression of pathogenic protein aggregation in neurodegenerative diseases such as Alzheimer's (AD) and Parkinson's disease (PD) are attracting increasing interest. Eisenberg, Baker, and co‐workers presented a strategy aimed at crafting peptides with structures that allow them to form steric zippers with the aggregation‐prone regions (APRs) of disease‐associated proteins like Aβ42, Tau, or Transthyretin, in order to block the elongation step of the amyloid polymerization reaction (patent US20100204085A1). The idea was portrayed with D‐TLKIVW (all D stereoisomer residues) peptide, which effectively delayed Tau K12 aggregation in a concentration‐dependent manner. Santos and co‐workers described how bacterial (PSMα3) and human (LL‐37) peptides can arrest α‐synuclein amyloid formation by binding selectively to toxic oligomers without disrupting the functional monomeric protein. This nanomolar interaction significantly mitigated the oligomers' neurotoxicity [89]. They identified the sequential/structural determinants of such activity to be a synergy of α‐helical conformation, cationic, and amphipathic characters. The authors used this concept to develop αSynPep‐DB, a dedicated and structurally oriented database of >100 biogenic peptides with the potential to block α‐synuclein amyloid aggregation and mitigate its cellular damage. In addition, they devised an accompanying algorithm for weighting these features on top of novel peptide sequences [90].

On the flip side, peptides can be theoretically designed to provoke the aggregation of pivotal proteins within pathogenic agents or cancer cells, potentially leading to a knockout effect or proteostasis disruption [91, 92, 93]. Initial attempts using linear peptides resembling the target proteins had little impact on the viability of malaria and leishmaniasis‐causing parasites [94, 95]. However, subsequent research by Switchlab identified key characteristics necessary for these aggregation‐inducing peptides (Pept‐ins) to target proteins effectively. The optimal design strategy involved a tandem repeat of two identical APRs flanked by solubilizing arginine residues and connected by a single proline. These peptides maintained a disordered structure in isolation yet exhibited the ability to form cross‐β supramolecular amyloid architectures upon encountering the target protein sequence [96].

### Antimicrobial peptides (AMPs)

The discovery of melittin, an antibacterial and antifungal peptide from bee venom in 1952 [6] marked the beginning of the identification of over 4000 AMPs in fungi, protists, animals, plants, archaea, or bacteria [97]. These peptides provide a natural defense mechanism to keep microbial populations under control, combat pathogenic infections, or avoid colonization of competing bacterial populations. As a general trend, they exhibit broad‐spectrum antimicrobial activities, killing or inhibiting the growth of Gram‐negative and Gram‐positive bacteria, fungi, and protozoa. AMPs, therefore, emerge as an alternative approach to fight microorganism infections, with promising application prospects in medicine, veterinary, agriculture, and the food industry [98, 99]. Since their mode of action diverges from conventional antibiotics, AMPs are becoming increasingly relevant in confronting multidrug‐resistant bacterial strains.

AMPs mainly comprise cationic and amphipathic peptides where the basic residues and the hydrophobic groups segregate spatially into amphiphilic structures (Fig. 1 – LL37). This amphipathic character allows them to interact with microbial membranes, which are rich in negatively charged molecules such as phospholipids. These contacts trigger membrane disruption by membrane embedding and subsequent pore formation [100]. Cyclic AMPs illustrate the tight connection between structure and function, as their activity depends on both the cycle size and the presence of exposed aromatic motifs [101].

Different AMPs have been shown to spontaneously assemble into amyloid structures. These fibrils exhibit a striking polymorphism since they can form typical cross‐β but also cross‐α motifs, with secondary structural transitions occurring in the fibril form upon contact with lipids or detergents [102, 103]. This highlights how the innate capacity to self‐assemble into amyloid macromolecular structures enables organisms to dynamically regulate AMPs between stable storage as a β‐assembly and an active cytotoxic α‐state.

The potential of AMPs to combat resistant bacteria prompted the development of various bioinformatics resources that focus on different aspects of these peptides. DRAMP [53] and DBAASP [26] are manually curated databases listing thousands of peer‐reviewed research articles. Additionally, DRAMP includes peptides from patents. Both databases consider the structural influence on AMP function by linking available PDB entries, with DBAAS even including molecular dynamic trajectories of peptides in solution. However, the predictive model used by DBAASP is limited as it only utilizes the sequential information of amino acids.

### Anticancer peptides (ACPs)

Peptides possessing selective and cytotoxic properties against one or more types of cancer cells are known as anticancer peptides. ACPs offer distinct advantages compared to antibodies and small molecules since they combine a relatively high selectivity, high penetration and easy modifiability [104], rendering them a still underexplored class of biotherapeutics to complement chemotherapies that employ organic molecules and biologics like antibodies, nucleic acids, and vaccines.

ACPs are typically helical peptides with an amphiphilic arrangement of their positive charges and hydrophobic residues [105]. These attributes mirror those of AMPs, with several peptides displaying dual activity as both anticancer and antimicrobial agents [106].

ACPs often exploit the significant structural abnormalities of cancerous cell membranes, including increased external negative charge, heightened fluidity provoked by the loss of bilayer asymmetry and increased microvilli exposure, resulting in an enlarged cell surface area [107]. This is why ACP membranolytic activity toward malignant cells has minimal effect on their healthy counterparts. Moreover, as ACPs exert their cytotoxic effects at the membrane level and over different intracellular targets (including mitochondrial membrane), they display reduced propensity to resistance emergence compared to single‐target chemotherapeutics. One example of this kind of drug is LTX‐315, a chemical derivative of Bovine lactoferricin, which has shown significant oncolytic activity in cell cultures and mouse models [108] and is currently undergoing phase III clinical trials. Besides naturally encoded ACPs, peptides released by protein hydrolysis during digestion have also shown antiproliferative activities in different across various cancer cell types [109].

Different endeavors have been undertaken to rationally develop peptides with specific conformations aimed at targeting oncogenes and tumor suppressor genes. Fersht and co‐workers derived peptides from the loops of 53BP2 binding protein to p53 tumor suppressor. The nonapeptide CDB3 stabilizes p53's native conformation and enables sequence‐specific DNA recognition, even restoring the DNA‐binding capacity of highly destabilized and oncogenic P53 mutants [110]. Also, different peptides have been designed to target the Myc oncogene, deregulated in most (and perhaps all) human cancers [111]. Myc and its obligatory partner Max are mostly intrinsically disordered proteins that undergo coupled folding and binding [112]. The resulting heterodimerization allows DNA binding and regulation of specific gene transcription [113]. At least two peptides are undergoing clinical trials, H1, corresponding to the Helix1–14 residues of Myc fused to an internalization peptide, and Oncomyc, a 91 residue Myc‐derived peptide. Both peptides have been developed to allow dimerization but impede DNA binding, therefore blocking translation and cell proliferation. While Oncomyc has shown efficacy when used alone, H1 could support chemotherapeutics [114]. A crucial functional requirement for both peptides is their ability to gain an α‐helical conformation and form a leucine zipper with Myc and or Max [115].

Almost 3500 ACPs with experimentally validated activity can be found in the database of anticancer peptides CancerPPD [116], including synthetic, chemically modified and naturally occurring ACPs. The predicted tertiary structures for all peptides are displayed in the database, signaling a growing awareness among researchers regarding the critical role of peptide conformations in anticancer activities.

## Discussion and conclusions

Peptides are biological entities that lay in the interface of small molecules and proteins [117]. While small organic molecules, and to a lesser extent replacement enzymes and antibody therapies, have historically been the preferential candidates for drug development, the distinct advantages offered by peptide‐based drugs should not be overlooked. Throughout this review, we have stressed that, much like proteins, the conformation of peptides is crucial for their function, stability, solubility, and target engagement.

Incorporating this structural information, however, poses a significant challenge due to limitations in existing data formats. The “FASTA” notation, commonly used for proteins and peptides, is limited to the primary structure, i.e., the amino acidic sequence [118]. While variants exist to extend this notation to PTMs, they fall short when describing other characteristics of peptides, such as cyclic structures or non‐amino acidic components [119]. Conversely, solutions designed for small molecules, like SMILES and SELFIES, capture all chemical and structural features of peptides, including the presence of chemical groups, cycles, or disulfide bonds, but its atom‐focused notation obscures the amino acidic nature of the peptide sequence [120].

The dualistic nature of peptides manifests as data gaps in repositories, with several aspects of peptide annotation, mostly related to their structure, being systematically underrepresented. For example, even though cycles are essential for the proper function of polymyxins, they are still not represented in a machine‐readable form in most AMP databases. This lack of information limits the capabilities of machine learning models for peptide function prediction, as they focus primarily on the sequence of amino acids [19], which is not the only informative feature.

Peptidic drugs have emerged as valuable therapeutic entities, offering versatility in treatment options, whether administered alone or in combination with non‐peptidic molecules. With this review, we wanted to illustrate how the application of peptides for different therapeutic purposes heavily relies on their conformational traits. Other applications not discussed in this review include immunomodulation, antiviral treatment, or weight loss. Ultimately, we aspire to catalyze integrative efforts within the theoretical and experimental communities to consolidate available functional and structural peptide data into accessible, computationally tractable repositories. Such initiatives would empower academia and industry alike to harness the vast potential of naturally occurring and synthetic peptides. With the rise of artificial intelligence is a compelling reason to prioritize this crucial undertaking. By building a comprehensive data infrastructure with unified structural annotation criteria, we can accelerate the development of more effective and targeted peptide‐based therapies.

## Conflict of interest

Salvador Ventura holds a European Patent related to the peptide modifiers of protein aggregation.

## Author contributions

VI and MB drafted the manuscript. OB designed the figures. All authors participated in the study's conceptualization and revised and approved the manuscript.
